# Regional variations and trends in liver transplantation practices across Europe

**DOI:** 10.1016/j.jhepr.2025.101424

**Published:** 2025-04-11

**Authors:** Tommaso Di Maira, Valérie Cailliez, Beatriz Domínguez-Gil, Beatriz Mahíllo, Marina Álvarez, Luca Saverio Belli, René Adam, Constantino Fondevila, Giacomo Germani, Hermien Hartog, Marina Berenguer

**Affiliations:** 1 Liver Transplantation and Hepatology Unit, Hospital Universitari I Politècnic La Fe, Valencia, Spain; 2 CIBERehd, Instituto de Salud Carlos III, Madrid, Spain, IIS La Fe, Valencia, Spain; 3 ELTR, Assistance Publique-Hôpitaux de Paris, Hôpital Paul Brousse, Centre Hepato-Biliaire, Université Paris-Sud, Villejuif, France; 4 Organización Nacional de Trasplantes (ONT), Madrid, Spain; 5 Department of Hepatology and Gastroenterology, Niguarda Hospital, Milan, Italy; 6 Hepatopancreatobiliary Surgery & Transplantation, General & Digestive Surgery Service, Hospital Universitario La Paz, IdiPAZ, CIBERehd, Madrid, Spain; 7 Multivisceral Transplant Unit, Department of Surgery, Oncology and Gastroenterology, Padua University Hospital, Padua, Italy; 8 Department of Surgery, Section of HPB & Liver Transplantation, University Medical Center Groningen, Groningen, The Netherlands

**Keywords:** Organ allocation, Waiting list mortality, Alcohol-related liver disease, Metabolically associated steatohepatitis

## Abstract

**Background & Aims:**

Liver transplantation (LT) is a live-saving therapy for patients with end-stage liver disease, but demand exceeds supply, leading to waiting list (WL) mortality. This study reviews LT practices and trends in Europe to identify potential policies for improving outcomes.

**Methods:**

Data were extracted from the European Liver Transplant Registry and the Global Observatory on Donation and Transplantation. Countries were categorized into Eastern (EEC), Mediterranean (MEC), and Northern European (NEC). We analyzed LT indications, recipient and donor age, transplant type, and WL outcomes from 2012 to 2022.

**Results:**

Etiology of LT differed across regions: HBV cirrhosis predominated in EEC (37.3%), whereas alcohol-related liver disease was more frequent in NEC (41.8%) and MEC (49.1%). Metabolic-dysfunction associated steatotic liver disease increased across Europe, particularly in NEC. Recipient age has risen, with 40% aged ≥60 years in MEC *vs.* 20% in EEC. Donor age and type also varied: EEC relies on younger donors (<50 years, 70%), whereas MEC expanded criteria to include donors ≥60 years (50%). Donation after circulatory determination of death increased by 30%, particularly in NEC and MEC, but remains rare in EEC. Model for end-stage liver disease scores at LT decreased, with 30% scoring >21 in 2021 *vs.* 50% in 2012. WL mortality declined by 10% since 2015, although large inter-country variability persists.

**Conclusions:**

LT practices in Europe are highly heterogeneous. Regional disparities in recipient profiles, donor characteristics, and transplant modalities reflect varying policies and healthcare capacities. Expanding donor criteria and harmonizing allocation systems are required to reduce WL mortality and improve access to LT across Europe.

**Impact and implications:**

This study provides a comprehensive analysis of liver transplantation practices across Europe, highlighting significant regional disparities in donor criteria, allocation systems, and transplant outcomes. By identifying trends such as the expansion of Donation after Circulatory Determination of Death programs and the prioritization of model for end-stage liver disease ≥30 policies, these findings underscore the critical need for harmonized strategies to reduce waiting list mortality and improve access to transplantation. The results are particularly relevant for policymakers and healthcare administrators seeking to optimize liver transplant systems, and for clinicians aiming to adopt best practices from high-performing regions. Practical applications include refining allocation policies, expanding donor pools, and addressing regional inequalities, all while considering the limitations posed by diverse healthcare infrastructures and socioeconomic factors.

## Highlights

• Heterogeneity in liver transplantation practices persists, with high WL mortality in many regions.
• Expanding donor criteria and refined allocation systems improved equity and LT outcomes.
• Countries with higher UHC indices and GDP had better LT accessibility and lower WL mortality.
• Policy changes are needed to balance urgency and fairness, especially for high MELD patients.
• Strengthening deceased donation programs and healthcare infrastructure can bridge efficiency gaps.

## Graphical abstract

**Figure:**
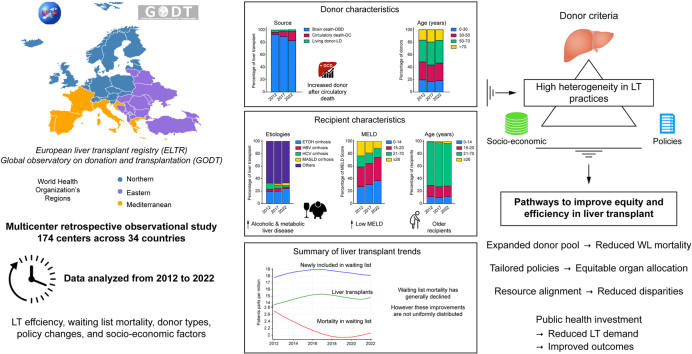

## Introduction

Liver transplantation (LT) stands as a crucial intervention for thousands of patients with end-stage liver disease globally. However, in most areas of the world with an LT program, the demand for liver organs consistently surpasses the available supply, leading to significant mortality rates among those awaiting transplantation.^1^ The scarcity of organs has led to the development of intricate allocation rules based on disease severity, such as the model for end-stage liver disease (MELD) scoring system, aimed at ensuring an equitable distribution among a diverse patient population.^2^

Over the years, the burden of liver disease, particularly cirrhosis, has surged worldwide. Deaths attributed to cirrhosis have increased, underscoring the pressing need for LT.^3^ Globally, the rates of LT have risen, albeit with substantial disparities across regions, both in volume and donor types. Although deceased donor LT predominates in the Western world, living donor LT (LDLT) remains predominant in many Asian countries, the Middle East and Turkey, reflecting regional variations in transplant practices.^4^

The etiologies driving cirrhosis and LT indications exhibit notable regional differences and temporal shifts. Notably, the advent of direct-acting antivirals (DAAs) has led to a decline in hepatitis C virus (HCV)-related LT indications in the US and Europe.^5^ Conversely, alcohol-associated liver disease (ALD) has emerged as a prominent indication, particularly in Europe and North America, with a concerning rise in incidence after the COVID-19 pandemic.^6^ Moreover, the landscape of LT has been shaped by demographic shifts, with older recipients becoming increasingly common.^1^^,^^7^ Additionally, the rising prevalence of metabolic-dysfunction associated steatotic liver disease (MASLD), closely linked to the obesity epidemic, presents a growing challenge. MASLD, with or without hepatocellular carcinoma (HCC), has become a leading indication for LT, particularly among older patients and women.^8^

Challenges persist regarding donor characteristics, with an increasing reliance on older donors, donors declared dead by circulatory criteria, and a rising proportion of steatotic grafts, posing concerns for graft outcomes.^1^^,^^7^ Furthermore, disparities in waitlist mortality and access to transplantation highlight the need for improved allocation strategies, especially for older candidates.^9^

In light of these trends, comprehensive strategies are imperative to address the evolving landscape of LT, including optimizing donor selection, refining allocation policies, and enhancing post-transplant outcomes. By leveraging insights from data collected across Europe, tailored approaches can be devised to mitigate the challenges and ensure equitable access to life-saving transplantation services, and overall to enhance liver health in the general population.

## Materials and methods

### Study origin and objectives

This study originates from discussions held during the 30th anniversary event of the European Liver and Intestine Association (ELITA) and the European Liver Transplant Registry (ELTR) in April 2024 in Madrid (Spain). At this summit, the authors were tasked with reviewing the changes in LT practices across Europe, focusing on techniques, recipient profiles, and donor characteristics. This paper summarizes our findings and provides insights into the conclusions drawn and potential solutions proposed based on this analysis.

### Data source

Data on adult LT were obtained from:

• **ELTR:** The ELTR has prospectively collected LT data from 174 centers across 32 European countries since 1986. It provides comprehensive information on activities within Europe. The main variables extracted from this source were: LT etiologies, number of transplants (N), European areas, population, total liver transplants, LT (per million population [pmp]), percentage of LT indications by country, percentage of LT indications in European areas, HCC (liver cancer), malignancy, cirrhosis etiology, primary disease details, MELD score, recipient age, donor age, recipient sex, sex frequency, MELD percentage, total HCC patients.
• **Global Observatory on Donation and Transplantation (GODT):** This source is the most comprehensive repository of global organ donation and transplantation data, developed through collaboration between the World Health Organization (WHO) and the Spanish Organización Nacional de Trasplantes (ONT). For this study, only European data were used, aligning with the regional focus of our research (https://www.transplant-observatory.org/export-database/). GODT collects data from 194 WHO member states through an annual questionnaire distributed to health authorities and designated individuals, gathering information on organizational structures, legislative aspects, and transplantation activities. The key variables extracted from GODT include: total population, types of LT (donation after brain death [DBD]; donation after circulatory determination of death [DCDD]; living donor [LTLD]), transplantability rate, total liver transplants, waiting list (WL) metrics (newly included, ever active, active at year-end, deaths, pmp), LT pmp, mortality rate, liver disease death metrics (liver cancer, cirrhosis, general liver disease).
• **National LT Organizations and Health Authorities:** Additional data were integrated from public sources provided by major LT organizations and European national health authorities, covering transplant activities, allocation policies, and organ donation practices (see Table S4). The key variables extracted were: regional classification, gross domestic product (GDP) *per capita* (2023), life expectancy (2022), intensive care unit (ICU) and hospital beds *per capita*, alcohol consumption *per capita*, Universal Health Coverage (UHC) index (2021), health expenditure (2012, 2022), change in health expenditure (absolute, relative), Total Health System index.

### Data integration and verification

We merged data from ELTR and GODT on a country-by-country and outcome-specific basis using unique identifiers to create a comprehensive dataset of European LT activities. Each data point was associated with a specific country and outcome to ensure unique identification across both databases. When the same data were available in both databases, discrepancies were reconciled by referring to the most recent or comprehensive entry. For countries or outcomes where data were missing in one database but available in the other, we integrated the available data without duplication.

The study focused on European countries that were categorized in three regions:

• **Eastern Europe** (Azerbaijan, Belarus, Bulgaria, Georgia, Hungary, Iran, Lithuania, Romania, Serbia, Turkey);
• **Mediterranean Europe** (Croatia, France, Greece, Italy, Portugal, Slovenia, Spain);
• **Northern Europe** (Austria, Belgium, Czech Republic, Denmark, Estonia, Finland, Germany, Ireland, Latvia, Luxembourg, The Netherlands, Norway, Poland, Slovakia, Sweden, Switzerland, UK).

### Statistical analysis

Data are presented as percentages and frequencies. The distribution of variables was assessed by the Shapiro–Wilk test and visual inspection with histograms, and missing data were handled using listwise deletion. We calculated 95% CIs for precision, and statistical tests such as *t* tests or ANOVA were used for group comparisons, with adjustments for multiple comparisons where applicable.

Mixed-effects models analyzed longitudinal data, accounting for fixed and random effects, to evaluate changes in LT activity, trends in oncologic indications, and the influence of healthcare and socioeconomic factors. Generalized linear mixed models assessed sex-related differences in LT, considering factors such as recipient age, transplant indication, and region. Linear regression models estimated annual changes in LT activity, trends in oncologic indications, and associations between gender representation and demographic factors.

Graphical methods, including locally weighted scatterplot smoothing (LOWESS), were used to visualize temporal trends in LT activity, WL inclusions, WL mortality, oncologic indications, and the impact of policy changes. A forest plot assessed the effects of different variables on gender differences in LT. Principal component analysis was used to reduce dimensionality in the analysis of healthcare and socioeconomic factors.

All analyses were conducted using Stata version 18 software (StataCorp LCC, College Station, TX, USA, 2023), and statistical significance was set at *p* <0.05.

### Compliance with ethical standards

This research was conducted in accordance with both the Declarations of Helsinki and Istanbul. The ELTR ensures GDPR compliance by pseudonymizing patient data and obtaining informed consent in line with GDPR Article 9.2 (i) and (j). The French National Ethics Committee (Commission Nationale de L'Informatique et des Libertés) approves data acquisition. Data are only shared with third parties following approval from the ELTR/ELITA scientific board and under a formal Data Processing Agreement to ensure confidentiality and restricted use (http://www.eltr.org). GODT aligns with international data protection standards by ensuring that data privacy and protection are maintained, involving only verified health authorities in the data provision process. Data from GODT are collected and maintained jointly by ONT and WHO, guaranteeing accuracy, reliability, and adherence to ethical guidelines (https://www.transplant-observatory.org).

## Results

### The demand of LT in Europe

**Fig. 1:**
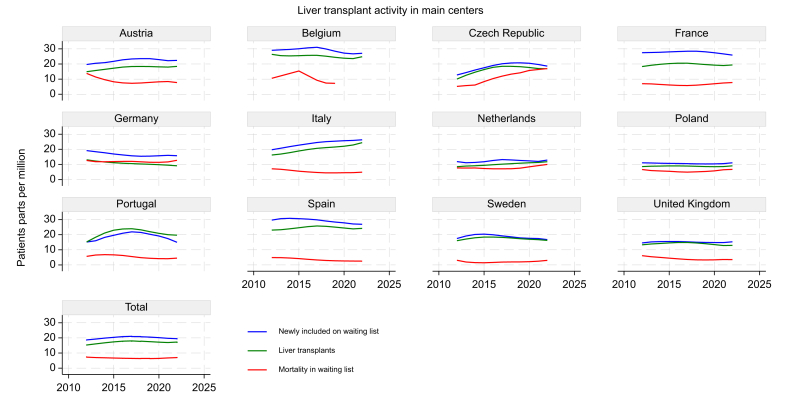
Trends in liver transplant activity in Main European countries. The figure displays the number of patients per million for three key metrics: newly included on the waiting list (blue line), liver transplants performed (green line), and mortality on the waiting list (red line), across European countries. Trends visualized using locally weighted scatterplot smoothing (LOWESS).

During the past 10 years, the demand of LT across European countries was noticeably heterogeneous (Fig. 1). Austria, Czech Republic, Italy, The Netherlands, and Switzerland saw consistent increases in LT activity with mean annual percentage increases of 3.3%, 5.6%, 4.2%, 6.0%, and 4.3% (*p* = 0.006, *p* = 0.041, *p* <0.001, *p* <0.001, *p* = 0.006), respectively. In terms of newly included patients on the WL, Czech Republic, Italy, and Switzerland showed significant increases of 3.9%, 3.3%, and 3.9% (*p* = 0.008, *p* <0.001, *p* = 0.001), respectively, whereas Austria showed a positive trend of 2.8% (*p* = 0.063).

Countries such as Spain showed a slight positive trend in LT activity (0.9%, *p* = 0.617) but a significant decline in new WL registrants (-20.8%, *p* = 0.016). In contrast, Germany experienced decreases in both LT activity and WL inclusion (-3.6%, *p* <0.001 and -1.9%, *p* = 0.112).

Other countries with significant changes in LT activity but not included in Fig. 1 because of lower LT activity were Lithuania and Romania, which showed significant increases in LT activity (11.6%, *p* = 0.029; 3.5%, *p* = 0.023, respectively), whereas Croatia showed a significant decrease (-3.2%, *p* = 0.024).

Although the progressive elimination of HCV may have played a role in these trends,^10^ it is important to understand the factors behind these heterogeneous WL numbers. Unfortunately, there is no information available on the referral process to LT centers, for instance, whether patients with some potential indications or characteristics are not properly referred (*e.g.* large HCC, acute-on-chronic liver failure 3 [ACLF 3], severe ALD, among others).

The assessment of LT programs’ efficiency across the countries in 2022, gauged by the percentage of patients active on the WL who received a transplant within a given year, shows that a substantial number of countries have a robust transplant program (Table 1). Notably, Austria, Croatia, Denmark, Finland, Ireland, Italy, The Netherlands, Poland, Portugal, Slovakia, Slovenia, Spain, Sweden, and the UK managed to serve a significant portion of their patients on the WL, with percentages over 50–70%, indicating effective organ allocation and transplant processes. However, some countries displayed less efficiency, potentially because of organ shortage or other LT program issues. Approximately half of the patients on the WL received an LT in 2022. This metric should be interpreted cautiously, as it does not represent the true probability of transplantation, as patients waitlisted in one year may receive the organ in subsequent years. Additionally, system efficiency could be over- or underestimated depending on WL inclusion criteria.

**Table 1:** 2022 European liver transplant overview: waiting list activity and transplantation outcomes by country.

Country	Ever active on the WL (n)	Ever active on the WL (pmp)	LT (n)	LT (pmp)	Active on WL who received LT (%)
Austria	295	32.4	169	18.6	57
Azerbaijan	NA	NA	NA	NA	NA
Belarus	NA	NA	NA	NA	NA
Belgium	NA	NA	297	25.4	NA
Bulgaria	66	9.7	6	0.9	9
Croatia	165	40.2	89	21.7	54
Czech Republic	NA	NA	180	16.8	NA
Denmark	85	14.7	46	7.9	54
Estonia	21	16.2	9	6.9	43
Finland	78	13.9	62	11.1	79
France	3,219	49.1	1,294	19.7	40
Germany	2,144	25.6	748	8.9	35
Greece	145	14.1	36	3.5	25
Hungary	153	15.9	67	7.0	44
Iran	NA	NA	NA	NA	NA
Ireland	89	17.8	51	10.2	57
Italy	2,620	43.4	1,479	24.5	56
Latvia	8	4.4	3	1.7	38
Lithuania	147	54.4	32	11.9	22
Luxembourg	6	NA	NA	NA	NA
The Netherlands	296	17.2	211	12.3	71
Norway	NA	NA	NA	NA	NA
Poland	606	16.1	362	9.6	60
Portugal	258	25.5	202	20.0	78
Romania	467	24.6	75	3.9	16
Slovakia	72	13.1	43	7.8	60
Slovenia	33	15.7	18	8.6	55
Spain	1,656	35.5	1,159	24.8	70
Sweden	257	25.2	166	16.3	65
Switzerland	NA	NA	NA	NA	NA
Turkey	NA	NA	NA	NA	NA
UK	1,836	26.8	922	13.5	50
**Average**	**167.5**	**23.4**	**172.5**	**13.0**	**55**

Data include the number of individuals ever active on the WL and those receiving LT, presented as both absolute numbers and pmp. The percentage of WL patients who received LT is also provided. LT, NA: not available, liver transplantation; pmp, per million population; WL, waiting list.

### Donor type

DCDD LT activities increased by 30% in the European region, particularly in Northen and Mediterranean regions, driven by expanded donor criteria and the inclusion of older and marginal donors in countries like Spain, Italy, and The Netherlands (Fig. S1). These changes helped address organ scarcity, leading to shorter WL times and reduced WL mortality. However, disparities in DCDD practices across the European sub-regions highlight the need for a deeper analysis of these variations. Although DCDD was well-established in Northern Europe a decade ago, Mediterranean countries have seen more recent expansion. Conversely, DCDD has not been developed in Eastern Europe, where LDLT remains more prevalent, particularly in Turkey. Insights into these regional differences are crucial for shaping future LR policies and practices.

### Donor age

**Fig. 2:**
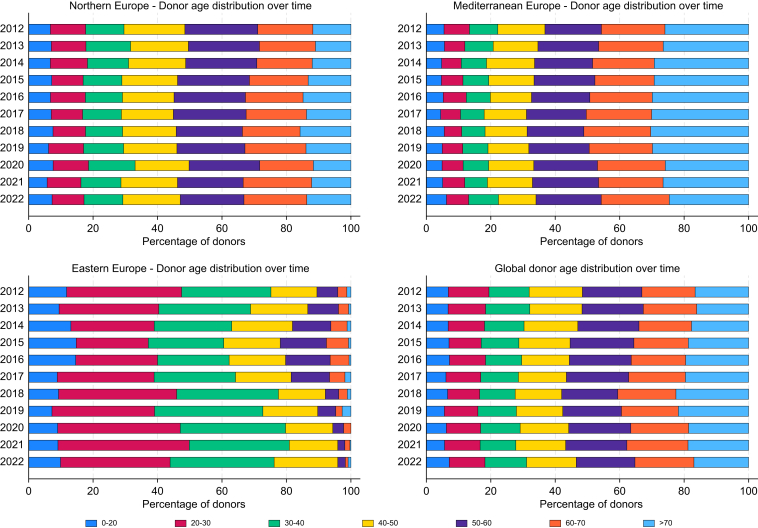
Evolution of donor age distribution in Europe. The figure displays the age distribution of liver donors over time in Northern, Mediterranean, and Eastern Europe, and the overall trend in Europe. The age groups range from 0–20 to >70 years, showing changes in the reliance on younger *vs.* older donors across regions. Descriptive data displayed as stacked bar charts of donor age distribution over time.

Donor age varied across Europe, with Eastern Europe primarily relying on younger donors. In Eastern Europe, overall only about 12.3% of donors were aged 50 years or older, indicating a preference for younger donors. In contrast, Mediterranean countries have significantly expanded their donor pool, with the percentage of donors aged 50 years or older increasing from 63% to 66%, reflecting a broad inclusion of older individuals. with a relatively stable 53% of donors aged 50 years or older between 2012 and 2021. Expanding donor age criteria has proven to be a viable approach to addressing organ scarcity, especially with the increase in DCDD transplants. The use of older donors in Mediterranean countries could serve as a model for Northern and Eastern regions to shorten WL times and lower MELD scores at transplantation (Fig. 2).

### Recipient age

**Fig. 3:**
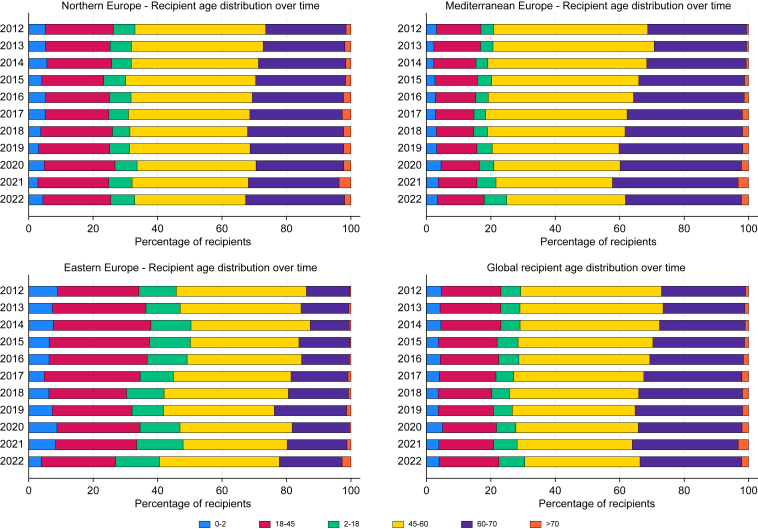
Evolution of liver transplant recipient age distribution in Europe. The figure illustrates the age distribution of liver transplant recipients over time in Northern, Mediterranean, and Eastern Europe, and overall trends across Europe. The age groups range from 0–2 to >70 years, highlighting changes in the recipient demographics over the last decade. Descriptive data displayed as stacked bar charts of recipient age distribution over time.

Globally, the proportion of LT recipients aged ≥60 years increased significantly, rising from 23.9% to 31% over the past decade. Despite this notable increase across the EU, especially among recipients aged 60–70 years – from 13.5% to 19.5% in Eastern Europe (coeff. 0.720, *p* = 0.003), 30.7% to 36.0% in Mediterranean Europe (coeff. 0.928, *p* <0.001), and 24.9% to 30.7% in Northern Europe (coeff. 0.402, *p* = 0.006) – a significant disparity remains in access to LT for older patients. The likelihood of receiving a transplant is nearly twice as high in Mediterranean countries compared with Eastern Europe (38.2% *vs.* 22.2%, respectively) (Fig. 3).

### Etiologies leading to LT

The etiologies leading to LT varied significantly across European regions. HBV cirrhosis was the leading cause in Eastern Europe, representing 37.3% of all cases, whereas ALD dominated in Northen and Mediterranean countries, with 41.8% and 49.1% of all cases, respectively (Fig. 2).

The evolution of etiologies leading to LT across Europe is also different. In Eastern countries, HBV decreased from 24% to 16% (coeff. 0.053, *p* = 0.685), whereas HCV significantly declined from 13% to 5% (coeff. -1.133, *p* < 0.001). In Northern countries, HCV decreased significantly from 7% to 1% (coeff. -0.728, *p* < 0.001). Similarly, in Mediterranean countries, HCV showed a significant reduction from 12% to 3% (coeff. -1.042, *p* <0.001).

In Northern countries, both ALD and MASLD (previously called non-alcoholic steatohepatitis or NASH) have grown substantially. ALD increased from 18% to 22% (coeff. 0.327, *p* = 0.001), and MASLD rose significantly from 1% to 8% (coeff. 0.688, *p* <0.001). Mediterranean countries have also experienced a significant increase in ALD, rising from 22% to 28% of all etiologies (coeff. 0.533, *p* = 0.019). Additionally, MASLD increased from 0.1% to 1.5% (coeff. 0.155, *p* <0.001).

**Fig. 4:**
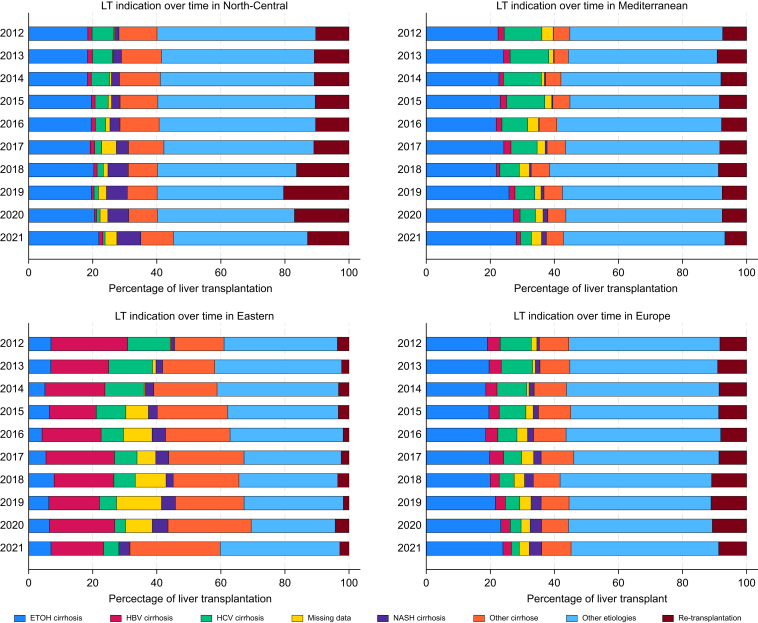
Evolution of liver transplant indications across Europe. The figure shows the changes in the etiologies leading to liver transplantation over time in North-Central, Mediterranean, and Eastern Europe, and overall trends across Europe. Etiologies include alcohol-related liver disease (ETOH cirrhosis), Hepatitis B (HBV) cirrhosis, Hepatitis C (HCV) cirrhosis, metabolic-dysfunction steatotic liver disease (NASH), and other causes. Descriptive data displayed as stacked bar charts of liver transplant indications over time.

These data suggest that robust welfare policies should be placed to reduce end-stage liver disease in preventable disorders with particular emphasis in preventing HBV and HCV infections in Eastern countries, alcohol misuse in Northern and Mediterranean countries and MASLD in Northern countries as emerging conditions (Fig. 4).

Considering the liver disease etiologies per country, the most relevant data are the increasing trend for alcohol cirrhosis, particularly in Austria (from 8% to 25%, coeff. 2.24, *p* = 0.001), Czech Republic (from 33% to 37%, coeff. 2.14, *p* = 0.043), Finland (from 18% to 28%, coeff. 1.13, *p* = 0.025), Hungary (from 9% to 19%, coeff. 3.55, *p* = 0.036), Italy (from 8% to 20%, coeff. 0.97, *p* = 0.002), and Spain (from 24% to 36%, coeff. 1.33, *p* <0.001). Other countries such as Croatia, The Netherlands, and Slovenia showed decreasing percentages.

The increasing MASLD etiology is appreciable in several countries showing significant positive trends, including Italy (from 0.27% to 6.64%, coeff. = 0.572, *p* = 0.001), Spain (from 0.09% to 1.62%, coeff. = 0.322, *p* = 0.027), Sweden (from 1.48% to 6.92%, coeff. = 0.539, *p* = 0.005), Switzerland (from 1.04% to 7.19%, coeff. = 0.560, *p* = 0.013), and the UK (from 4.10% to 9.20%, coeff. = 0.526, *p* <0.001). These low percentages may reflect underreporting or misclassification in other countries. However, the significant increase observed in many EU countries suggests that the trend may be more widespread.

### Trends in liver transplantation for oncologic indications

#### Hepatocellular carcinoma

Northern Europe experienced a significant decline in HCC-related liver transplants, with a consistent decrease from 16.1% in 2012 to 9.6% in 2021 (coeff. = -0.884, *p* <0.001). In contrast, Eastern and Mediterranean Europe showed stable rates of HCC-related transplants, with Eastern Europe changing from 5.7% to 8.5% (coeff. = -0.097, *p* = 0.190) and Mediterranean Europe from 25% to 26.3% (coeff. = 0.054, *p* = 0.380) (Fig. S3).

#### Non-HCC malignancies

The role of LT for non-HCC malignancies has expanded significantly, with several types becoming increasingly prevalent. Among the 392 non-HCC tumors that underwent LT as treatment, hepatic cholangiocellular carcinoma was the most frequent, representing 20.2% of non-HCC cases (n = 79), followed by other neuroendocrine tumors (17.1%, n = 67) and epithelioid hemangioendothelioma (15.1%, n = 59). Biliary tract carcinoma (Klatskin tumor) and carcinoid tumors also contributed, accounting for 14.0% (n = 55) and 11.0% (n = 43), respectively.

In Mediterranean Europe, the rate of LT as a result of non-HCC malignancies rose from 1.6% in 2012 to 1.9% in 2021, showing a significant upward trend (coeff. = 0.035, *p* <0.001). Conversely, Northern Europe saw a decline in such transplants, dropping from 1.8% in 2012 to 1.5% in 2021 (coeff. = -0.047, *p* <0.001). In Eastern Europe, no significant changes were noted, with rates remaining steady at 1.3% over the same period (coeff. = 0.006, *p* = 0.268). These trends emphasize the expanding role of LT in oncologic care beyond HCC, with a gradual but sustained rise in non-HCC indications across all regions (Fig. S4).

### Sex representation and LT

The analysis of sex representation in LT across Mediterranean, Eastern, and Northern Europe from 2012 to 2021 reveals a consistent higher male representation in all regions, with male representation ranging between ∼60% and 65% over the years. Despite male recipients consistently outnumbering female recipients, their representation is slightly decreasing in Northern Europe (coeff. -0.402, *p* = 0.013), whereas no significant change is observed in Eastern (coeff. -0.310, *p* = 0.321) and Mediterranean (coeff. 0.472, *p* = 0.431) European countries.

To further explore sex-related representations in LT, a multivariable analysis showed that middle-aged (45–60 years, coeff. 0.049, *p* = 0.185) and older recipient cohorts (>70 years, coeff. 0.038, *p* = 0.309) had a trend towards being male, whereas younger recipients aged between 18 and 45 years were more likely female (coeff. -0.106, *p* = 0.007). Moreover, males are more likely to receive LT for HCC (coeff. 0.152, *p* <0.001), which aligns with the higher incidence of HCC in men compared with women. For less common transplant indications such as non-HCC malignancies (coeff. -0.260, *p* <0.001) and other etiologies (coeff. -0.199, *p* <0.001), female recipients account for a higher proportion of cases.

Finally, when examining regional variations, Mediterranean (coeff. -0.472, *p* <0.001) and Northern Europe (coeff. -0.157, *p* = 0.003) show a stronger propensity for females to receive LTs compared with Eastern regions (Table S1 and Fig. S5). This trend may indicate regional factors influencing sex distribution in transplantation practices and warrants targeted investigation to understand the contributing factors. Although these findings are significant, it is important to consider the potential limitations and variability in data collection and reporting across the included countries.

### Evolution of biochemical MELD score

Interestingly, in contrast to US data, a trend to decreasing MELD scores is observed in European countries. In 2012, the proportion of patients with a MELD score >21 varied significantly across regions: 22.7% in Eastern Europe, 52.1% in Mediterranean Europe, and 35.9% in Northern Europe. By 2021, these percentages dropped to 19.1% of transplants in Eastern Europe, 23.2% in Mediterranean Europe, and 27.4% in Northern. Furthermore, only 13.1% of liver transplants performed in 2021 had a MELD score >26.

Additionally, the number of patients undergoing LT for presumably ‘exemption criteria’ with MELD scores <14 increased in some regions. In Eastern Europe, the proportion rose from 35.7% to 55.1% (coeff. 1.618, *p* = 0.089), whereas in Mediterranean Europe it increased from 18.0% to 35.7% (coeff. 1.422, *p* <0.001).

**Fig. 5:**
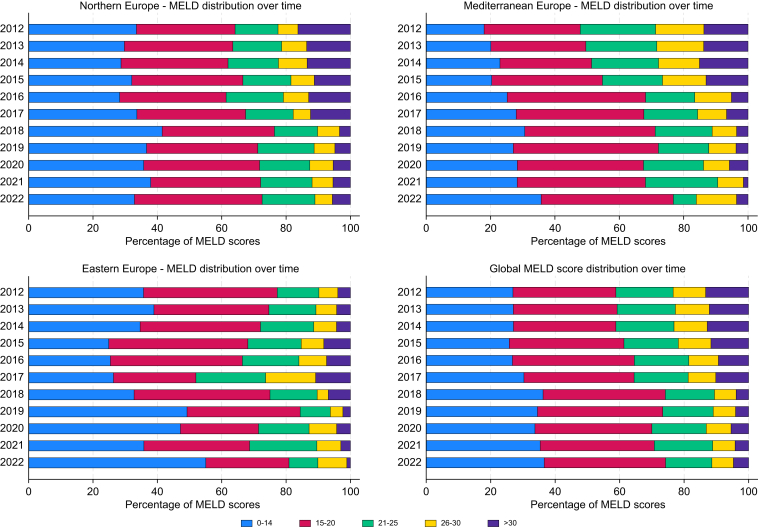
Evolution of biochemical MELD score distribution in Europe. The figure illustrates the changes in the distribution of MELD scores among liver transplant recipients over time across Northern, Mediterranean, and Eastern Europe, and Europe overall. MELD score categories are shown, ranging from 0–14 to >30, highlighting the shift towards lower MELD scores over the last decade. Descriptive data displayed as stacked bar charts of MELD score distribution over time.

The reasons for these findings are likely multifactorial, but potentially imply the limitations of the MELD system as a reliable tool to prioritize patients on the WL and the progressive increase of oncologic disease as an indication for LT (Fig. 5).

### Healthcare and socioeconomic factors influencing LT practices

The analysis of healthcare and socioeconomic variables across European countries (Table S2) highlighted significant factors influencing LT practices and efficiency. The UHC index was the only variable showing a statistically significant positive association with LT efficiency (coeff. = 1.299, *p* = 0.006), suggesting that greater healthcare coverage is associated with improved transplant opportunities.

Mixed-effects regression models using principal components revealed that general socioeconomic status and health investment, represented by Comp1 (including GDP, life expectancy, and healthcare expenditure), was positively associated with LT efficacy (coeff. = 5.312, *p* = 0.004). This indicates that countries with higher GDP and stronger healthcare investments tend to have higher LT success rates.

Conversely, Comp4, which included markers related to healthcare capacity (*e.g.* ICU beds, hospital beds, and healthcare expenditure), showed a significant negative association with LT efficacy (coeff. = -26.779, *p* = 0.024). This suggests that countries with substantial healthcare infrastructure but limited financial resources may face challenges in effectively utilizing these resources for LT, potentially hindering transplant opportunities (Table S3).

### Impact of policy changes on LT trends

The variability in LT practices across Europe during the study period was influenced by country-specific policy changes (Table S4). The expansion of donor criteria, such as the increased use of DCDD and inclusion of older donors, contributed to rising LT activity and reduced WL mortality, particularly in Northern and Mediterranean regions. Germany’s stricter allocation rules, introduced in 2013, initially led to decreased LT activity but improved transparency and fairness over time. The UK’s new allocation system in 2018, prioritizing patients with urgent medical needs, also contributed to reduced WL mortality. Centralized organ allocation systems in countries such as France and Poland improved coordination and maintained stable transplant activities despite the overall decline in MELD scores across Europe. Countries such as Italy and Spain also expanded donor criteria to include older donors, increasing the proportion of older LT recipients.

### Mortality on the WL

**Fig. 6:**
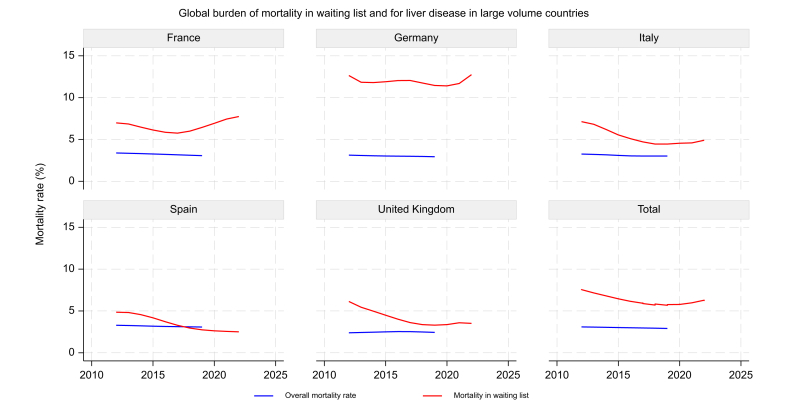
Global burden of mortality in the waiting list and from liver disease in large volume countries. The figure shows the trends in overall mortality rates from liver disease and mortality rates on the waiting list for liver transplantation in large volume countries (France, Germany, Italy, Spain, and the UK). The blue line represents the overall mortality rate, while the red line represents the mortality rate in the waiting list over time. Trends visualized using locally weighted scatterplot smoothing (LOWESS).

Overall, mortality rate on the WL decreased starting from 2015 observing a smoothed increase of LT and newly included patients (Fig. 6). The more evident reduction of mortality on the WL might be related to the advent of DAAs for HCV treatment and partially from the increasing LT activity. However, there is a significant heterogeneity across the countries, and this trend was interrupted by the COVID-19 pandemic, which led to increased mortality rates on the WL and reduced LT activities and new patient inclusions in 2020 and 2021. Considering the large volume countries, Germany shows a decreasing number of LTs and newly included patients on the WL, maintaining mortality rates unchanged over a decade (Fig. 1).

Interestingly, comparing the global burden of deaths from liver diseases treatable with LT and mortality on the WL in large countries, Germany showed an overall decrease of liver disease mortality that was not accompanied by a reduction on WL mortality. Contrarily, in the UK, the descending mortality on the WL is followed by a slight increase of liver disease mortality, potentially because of low referral to LT centers (Fig. 6).

## Discussion

This study reveals several key findings regarding LT practices across Europe, using data obtained from the ELTR and the GODT, with its potential limitations derived from the incompleteness and potential errors in data collection from such a large number of centers (n = 174) in 32 countries. Overall, the main conclusion from the different aspects analyzed refers to the heterogeneity of LT practices in Europe, in terms of the demand for LT, the use of DCDD and older donors, the access of older recipients to LT, or the LT indications. Although some countries such as Italy, the Czech Republic, and Switzerland have seen a consistent increase in LT activity and newly listed patients, others such as Germany have experienced a notable decrease in both metrics.

Factors contributing to these trends, such as the progressive elimination of HCV, warrant further investigation, particularly regarding the referral process to LT centers, the expansion of non-traditional donation including the use of DCDD or older donors, and the different impact of alcohol or obesity in the liver health of the population across the European countries.^3^ The variability in donor age across European countries, with Eastern Europe exhibiting notably lower donor ages compared with other regions, suggests that expanding donor age criteria, particularly with the inclusion of older donors as seen in Mediterranean countries, could help address the scarcity of available livers for transplantation and reduce WL times and mortality. Indeed, recent studies have demonstrated that the use of livers from older donors, including septuagenarian and octogenarian donors can achieve comparable outcomes after LT if careful selection methods are applied.^7^^,^^9^^,^^11^

General socioeconomic improvements, particularly reflected by the UHC index, positively influence LT efficiency. Countries with higher UHC scores, GDP, and health investments achieved better transplant opportunities, reflecting improved healthcare access and resource allocation. However, healthcare infrastructure alone, without stable economic support and efficient utilization, does not guarantee better outcomes. This highlights the need for aligning healthcare capacity with effective resource management to achieve successful LT opportunities.

The expansion of donor criteria, such as the inclusion of DCDD and older donors in countries such as Spain, Italy, and The Netherlands, contributed to the observed rise in LT activity, reduced WL times, and lower WL mortality. In contrast, stricter allocation regulations, such as those introduced in Germany and the UK, initially led to decreased transplant numbers but improved equity and transparency in the long term. These policy changes, along with the socioeconomic improvements in many European countries, have contributed to the heterogeneous landscape of LT practices across the continent.

Importantly, the phenotype of the LT recipient has also changed substantially. A substantial increase in recipient age is observed across all European countries over the last decade. However, there is notable inequity in access to LT for older patients, with Mediterranean countries showing higher rates of LT for this demographic compared with Eastern countries. This increase in age has been previously reported coinciding with the increased global life expectancy.^7^ Yet, it may also result from the recent lower pressure on WL in some countries, such as Spain and Italy, allowing an expansion of transplant indications. Older patients are more likely to have comorbidities, particularly cancer and cardiovascular events, as well as frailty compared with younger patients. This is associated with an increased risk of WL and post-transplant mortality. Indeed, short-term survival rates are equivalent for older and younger recipients; yet in the mid-long-term survival is inferior for the older individuals.^12^^,^^13^ A careful recipient selection with attention to functional status, physiologic organ reserve, and comorbidity is crucial in optimizing post-LT outcomes.^14^ In addition, incorporation of pre- and post-rehabilitation programs may help reducing the impact of frailty.[15], [16], [17], [18]

Together with age, indications are also heterogeneous across European countries with variations in prevalence and evolution over time. Although HBV cirrhosis predominates in Eastern countries, ALD is more prevalent in Northern and Mediterranean countries. These findings underscore the importance of welfare policies targeting preventable disorders to reduce the burden of end-stage liver disease. In particular, access to oral antivirals for both HBV and HCV, is the main driver to understand shifts in liver disease worldwide.^1^^,^^3^^,^^8^^,^^9^^,^^19^ In turn, ALD is one of the most prevalent causes of chronic liver disease across the globe.^3^ In 2017, nearly 123 million individuals were suspected of having alcohol-related cirrhosis.^3^^,^^20^ ALD is responsible for approximately 60% of hospitalizations from the complications of cirrhosis or ACLF and mortality is twice as high in patients with alcohol cirrhosis than in those with cirrhosis from other causes.^21^ Unfortunately, and differently from other chronic liver diseases, most patients with ALD are diagnosed at an advanced stage, when the disease becomes symptomatic.^22^ The expansion of ALD as an indication for LT requires an adequate collaboration from addiction specialists and psychiatrists–psychologists to address mental problems often present. MASLD is another etiology on the rise, particularly present in Northern European countries, similar to trends already present in the US.^1^^,^^8^ Introducing various alcohol and diet control measures in all European countries could reduce heterogeneity.^23^^,^^24^

The expansion of oncologic indications, including both HCC and non-HCC malignancies, has potentially influenced LT practices across Europe. Mediterranean Europe has shown a significant increase in non–HCC–related transplants, and a positive trend for HCC-related transplants, possibly because of stronger screening programs and proactive management of oncologic cases. In contrast, Northern Europe experienced declines in both HCC and non-HCC malignancy-related transplants, reflecting potentially different screening practices or shifts in eligibility criteria. Eastern Europe, meanwhile, showed no significant change in non–HCC–related transplants but had an increase, albeit non-significant, in HCC-related transplants. These changes reflect an ongoing effort to expand eligibility criteria and use LT as a curative treatment for certain oncologic conditions. This expansion of oncologic indications also affects MELD scores, especially with MELD exemptions, highlighting the need for a multidisciplinary approach for patient selection and post-transplant care to optimize outcomes for oncologic patients. The regional differences in transplant activity underscore the importance of harmonizing selection criteria and expanding access to LT for all eligible oncologic patients.

Although limitations in MELD data collection may explain our findings, our study identifies a trend of decreasing MELD scores in European countries over time. This suggests potential limitations of the MELD system as a reliable tool for prioritizing patients on the WL,^2^^,^^25^^,^^26^ particularly regarding patients with lower MELD scores but portal hypertension complications who undergo LT but might also reflect the increased in MELD exemption indications such as metastatic colorectal liver cancer, hilar cholangiocarcinoma or HCC, particularly in countries with lower WL pressure.^27^^,^^28^ Concretely, the expansion of oncologic indication for both HCC and non-HCC malignancies, has contributed to more patients undergoing transplantation at lower MELD scores as a result of these exception policies. Furthermore, the widespread use of DAAs has significantly reduced the progression of HCV-related liver disease, leading to fewer patients reaching advanced stages with high MELD scores. Simultaneously, the increase in MASLD has resulted in more patients being listed for transplantation with complications such as HCC rather than acute decompensation, often presenting with lower MELD scores. The expansion of donor criteria, including the use of older donors and DCDD, has improved organ availability, reducing waiting times and allowing patients with lower urgency to receive transplants sooner.

These factors, combined with allocation policies that may prioritize equitable access over medical urgency, contribute to the decreasing MELD scores at transplantation. This trend has important clinical implications, potentially leading to improved post-transplant outcomes attributable to patients being in better preoperative condition, but it also raises concerns about waitlist mortality for patients with higher MELD scores. Future policy adjustments should aim to balance these considerations to optimize outcomes for all patients.

One of the most relevant findings of this study refers to the mortality on the WL. Although there has been a global decrease in mortality rates on the WL since 2015, there is significant heterogeneity across countries. Factors such as the advent of new treatments for HCV and the increase in LT activity may contribute to this trend. However, some countries, such as Bulgaria and Croatia, show an increasing trend in mortality despite a stable LT activity, highlighting the need for further investigation into the underlying factors.

This study is limited by potential biases arising from variations in data collection across multiple centers and countries, potentially affecting data reliability. The diversity of healthcare systems and economic conditions among the countries also complicates generalizing the findings. Additionally, although advanced statistical models were used to adjust for confounding, residual confounding cannot be entirely ruled out. The use of aggregate data further limits the ability to analyze more nuanced patient-level factors that might impact LT outcomes.

## Conclusions

In essence, heterogeneity dominates LT activity in Europe, with WL mortality still excessive in many countries. Although expansion of LDLT may reduce the gap between donation and transplantation, an effort to expand deceased donation is likely necessary. Although this might indicate the need for changes in the respective local organizations, it may also reflect different attitudes towards the use of ‘less optimal organs’. The experiences of countries such as those shown by Italy and Spain, clearly demonstrate the potential for good outcomes with the use of such organs.^9^^,^^11^^,^^29^ The expansion of donor criteria and refined allocation systems possibly played a crucial role in shaping LT practices and reducing disparities in access across Europe during the study period, highlighting the importance of continued policy adjustments to improve outcomes.

This study also highlights the influence of healthcare and socioeconomic markers on LT efficiency. Countries with higher UHC indices, GDP *per capita*, and strong healthcare investments had better access to LT and lower WL mortality. In contrast, regions with extensive infrastructure but limited resources struggled to use them effectively. These findings underscore the need for policies that align healthcare capacity with efficient resource management to improve LT outcomes.

Overall, this study emphasizes the importance of understanding regional variations in LT practices and addressing disparities in access to transplantation. Insights derived from these findings can inform policy decisions and strategies aimed at improving the efficiency and accessibility of LT programs across Europe. Policymakers should consider aligning allocation systems to balance urgency and fairness ensuring patients with higher MELD scores are not disadvantaged. Additionally, efforts to strengthen healthcare infrastructure in economically constrained regions and improve access to resources can bridge gaps in LT efficiency.

Investments in training and infrastructure to support deceased donation programs, coupled with adopting successful practices from high-performing regions, should be prioritized. Focusing on comprehensive assessment tools for older patients and expanding public health measures to reduce preventable liver diseases, such as implementing vaccination programs and alcohol misuse prevention campaigns, could reduce LT demand and enhance LT outcomes. These strategies, combined with improved data collection and targeted research, are essential for optimizing LT practices and advancing liver health across Europe.

## Abbreviations

ACLF, acute-on-chronic liver failure; ALD, alcohol-related liver disease; DAAs, direct-acting antivirals; DBD, donation after brain death; DCDD, donation after circulatory determination of death; EEC, Eastern European countries; ELITA, European Liver and Intestine Transplant Association; ELTR, European Liver Transplant Registry; EMC, Eastern Mediterranean countries; GDP, gross domestic product; GODT, Global Observatory on Donation and Transplantation; HCC, hepatocellular carcinoma; ICU, intensive care unit; LDLT, living donor liver transplantation; LT, liver transplantation; MASLD, metabolic-dysfunction-associated steatotic liver disease; MEC, Mediterranean European countries; MELD, model for end-stage liver disease; NEC, Northern European countries; ONT, Organización Nacional de Trasplantes; pmp, per million population; UHC, Universal Health Coverage; WL, waiting list.

## Financial support

Funding was received from the Instituto de Salud Carlos III (Spain) and co-funded by European Union (grants PI23/00088 and INT24/00021 to MB; grant INT20/00061 to MB; and Juan Rodés Research grant JR18/00008 and grant PI20/00737 to TM); by the 10.13039/501100003359Generalitat Valenciana (Spain) (grant AICO/2021/035 to MB); by 10.13039/100006301CIBER (Consorcio Centro de Investigación Biomédica en Red, Spain) (grant CB06/04/0065); and by the Spanish Society of Liver Transplantation (SETH, Spain) (grant 2022/295 to MB). No sponsor had a role in the study design, the data collection, the analysis and interpretation of data, the writing of the paper or the decision to submit the article for publication.

## Authors’ contributions

Contributed to the development of the research idea: BDG, LSB, MB. Contributed to the design of the methodology: TM, BDG, LSB, MB. Collected data: TM, VC, BM, MÁ. Managed research data: TM. Performed data analysis and interpretation: TM, VC. Contributed to writing the manuscript: TM, MB. Contributed to the review and editing of the manuscript: VC, BDG, BM, MÁ, LSB, RA, CF, GG, HH, MB.

## Data availability statement

The data used in this study were mainly obtained from the European Liver Transplant Registry (ELTR). Access to these data is restricted to ELTR members or authorized personnel and may involve a fee. Interested researchers should contact ELTR via http://www.eltr.org or email eltr@ext.aphp.fr for further information. This study adhered to ELTR's data usage policies and ethical guidelines.

## Conflicts of interest

The authors declare no conflicts of interest that pertain to this work.

Please refer to the accompanying ICMJE disclosure forms for further details.

## References

[1] Terrault N.A., Francoz C., Berenguer M. (2023). Liver transplantation 2023: status report, current and future challenges. Clin Gastroenterol Hepatol.

[2] Tejedor M., Selzner N., Berenguer M. (2022). Are MELD and MELDNa still reliable tools to predict mortality on the liver transplant waiting list?. Transplantation.

[3] Devarbhavi H., Asrani S.K., Arab J.P. (2023). Global burden of liver disease: 2023 update. J Hepatol.

[4] Rela M., Rammohan A. (2021). Why are there so many liver transplants from living donors in Asia and so few in Europe and the US?. J Hepatol.

[5] Belli L.S., Duvoux C., Berenguer M. (2017). ELITA consensus statements on the use of DAAs in liver transplant candidates and recipients. J Hepatol.

[6] Cholankeril G., Goli K., Rana A. (2021). Impact of COVID-19 pandemic on liver transplantation and alcohol-associated liver disease in the USA. Hepatology.

[7] Durand F., Levitsky J., Cauchy F. (2019). Age and liver transplantation. J Hepatol.

[8] Younossi Z.M., Stepanova M., Al Shabeeb R. (2023). The changing epidemiology of adult liver transplantation in the United States in 2013-2022: the dominance of metabolic dysfunction-associated steatotic liver disease and alcohol-associated liver disease. Hepatol Commun.

[9] Trapero-Marugán M., Little E.C., Berenguer M. (2018). Stretching the boundaries for liver transplant in the 21st century. Lancet Gastroenterol Hepatol.

[10] Belli L.S., Perricone G., Adam R. (2018). Impact of DAAs on liver transplantation: major effects on the evolution of indications and results. An ELITA study based on the ELTR registry. J Hepatol.

[11] Díaz Jaime F., Berenguer M. (2017). Pushing the donor limits: deceased donor liver transplantation using organs from octogenarian donors. Liver Transpl.

[12] Su F., Yu L., Berry K. (2016). Aging of liver transplant registrants and recipients: trends and impact on waitlist outcomes, post-transplantation outcomes, and transplant-related survival benefit. Gastroenterology.

[13] Gómez Gavara C., Esposito F., Gurusamy K. (2019). Liver transplantation in elderly patients: a systematic review and first meta-analysis. HPB.

[14] Crespo G., Hessheimer A.J., Armstrong M.J. (2022). Which preoperative assessment modalities best identify patients who are suitable for enhanced recovery after liver transplantation? A systematic review of the literature and expert panel recommendations. Clin Transpl.

[15] Puchades L., Herreras J., Ibañez A. (2023). Waiting time dictates impact of frailty: a Spanish multicenter prospective study [published correction appears in JHEP Rep 2024;6:101097. JHEP Rep.

[16] Mina D.S., Tandon P., Kow A.W.C. (2022). The role of acute in-patient rehabilitation on short-term outcomes after liver transplantation: a systematic review of the literature and expert panel recommendations [published correction appears in Clin Transplant. Clin Transpl.

[17] Tandon P., Zanetto A., Piano S. (2023). Liver transplantation in the patient with physical frailty. J Hepatol.

[18] Pollok J.M., Tinguely P., Berenguer M. (2023). Enhanced recovery for liver transplantation: recommendations from the 2022 international liver transplantation society consensus conference (correction lancet gastroenterol hepatol 2023 Feb;8:117). Lancet Gastroenterol Hepatol.

[19] Duvoux C., Belli L.S., Fung J. (2021). 2020 position statement and recommendations of the European Liver and Intestine Transplantation Association (ELITA): management of hepatitis B virus-related infection before and after liver transplantation. Aliment Pharmacol Ther.

[20] GBD 2017 Cirrhosis Collaborators (2020). The global, regional, and national burden of cirrhosis by cause in 195 countries and territories, 1990-2017: a systematic analysis for the Global Burden of Disease Study 2017. Lancet Gastroenterol Hepatol.

[21] Singal A.K., Ahmed Z., Axley P. (2021). Hospitalizations for acute on chronic liver failure at academic compared to non-academic centers have higher mortality. Dig Dis Sci.

[22] Shah N.D., Ventura-Cots M., Abraldes J.G. (2019). Alcohol-related liver disease is rarely detected at early stages compared with liver diseases of other etiologies worldwide. Clin Gastroenterol Hepatol.

[23] Neufeld M., Bobrova A., Davletov K. (2021). Alcohol control policies in Former Soviet Union countries: a narrative review of three decades of policy changes and their apparent effects. Drug Alcohol Rev.

[24] Maharaj T., Angus C., Fitzgerald N. (2023). Impact of minimum unit pricing on alcohol-related hospital outcomes: systematic review [published correction appears in BMJ Open 2023;13:e065220corr1. BMJ Open.

[25] Paik J.M., Golabi P., Younossi Y. (2020). Changes in the global burden of chronic liver diseases from 2012 to 2017: the growing impact of NAFLD. Hepatology.

[26] Tejedor M., Neria F., De La Rosa G. (2024). Women are also disadvantaged in accessing transplant outside the United States: analysis of the Spanish Liver Transplantation Registry. Transpl Int.

[27] Krendl F.J., Bellotti R., Sapisochin G. (2023). Transplant oncology - current indications and strategies to advance the field [published correction appears in JHEP Rep 2024. JHEP Rep.

[28] Artzner T., Bernal W., Belli L.S. (2022). Location and allocation: inequity of access to liver transplantation for patients with severe acute-on-chronic liver failure in Europe. Liver Transpl.

[29] Romano P., Cano L., Pietrasz D. (2024). Liver transplantation from elderly donors (≥85 years old). Cancers (Basel).

