# Supplementary material for: Regional variations and trends in liver transplantation practices across Europe
Source: JHEP Rep. 2025 Apr 11;7(8):101424. doi: 10.1016/j.jhepr.2025.101424 (PMC12276449; doi:10.1016/j.jhepr.2025.101424)
Supplement: Multimedia component 1 [file mmc1.pdf]

Regional variations and trends in liver transplantation practices across Europe

Tommaso Di Maira, Valérie Cailliez, Beatriz Domínguez-Gil, Beatriz Mahíllo, Marina Álvarez, Luca Saverio Belli, René Adam, Constantino Fondevila, Giacomo Germani, Hermien Hartog, Marina Berenguer, for the European Liver and Intestine Transplant Association (ELITA)

Table of contents

Fig. S1…………………………………………………………………………………2

Fig. S2…………………………………………………………………………………3

Fig. S3…………………………………………………………………………………4

Fig. S4…………………………………………………………………………………5

Fig. S5…………………………………………………………………………………6

Fig. S6…………………………………………………………………………………7

Table S1……………………………………………………………………………....8

Table S2……………………………………………………………………………....9

Table S3…………………………………………………………………………….11

Table S4…………………………………………………………………………….12

Supplementary references………………………………………………………...14

Fig. S1 – Liver Transplant Activity by Donor Type

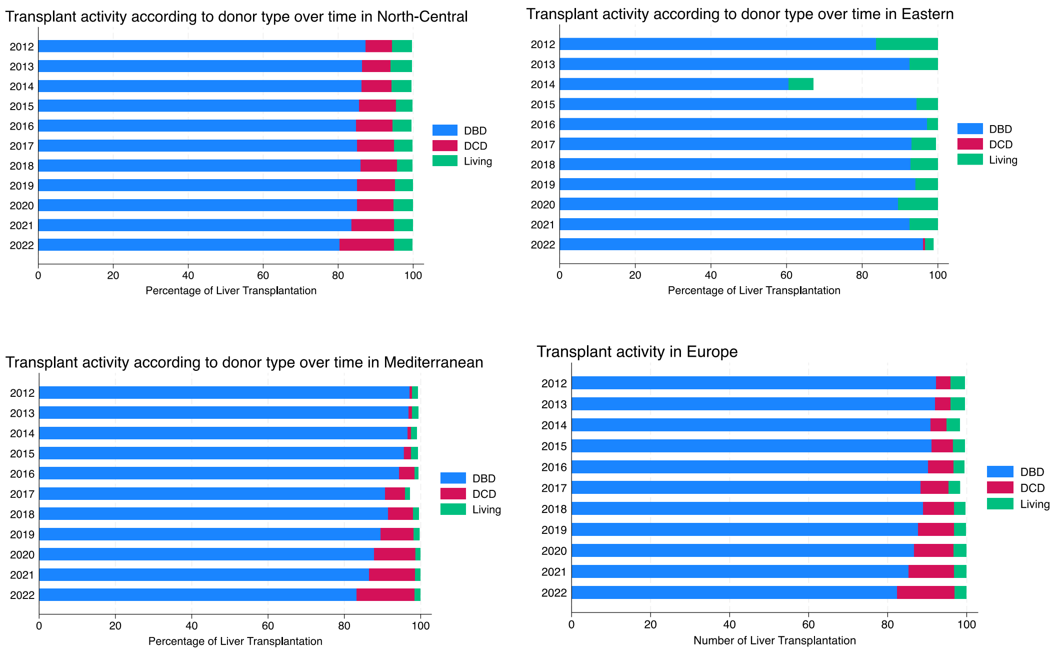

The figure shows the evolution of liver transplant activity according to donor type—Donation after Brain Death (DBD), Donation after Circulatory Death (DCD), and Living Donor Liver Transplantation (LDLT)—in North-Central, Eastern, and Mediterranean European regions, as well as Europe overall.

Fig. S2 – Liver Transplant Aetiologies by European Region

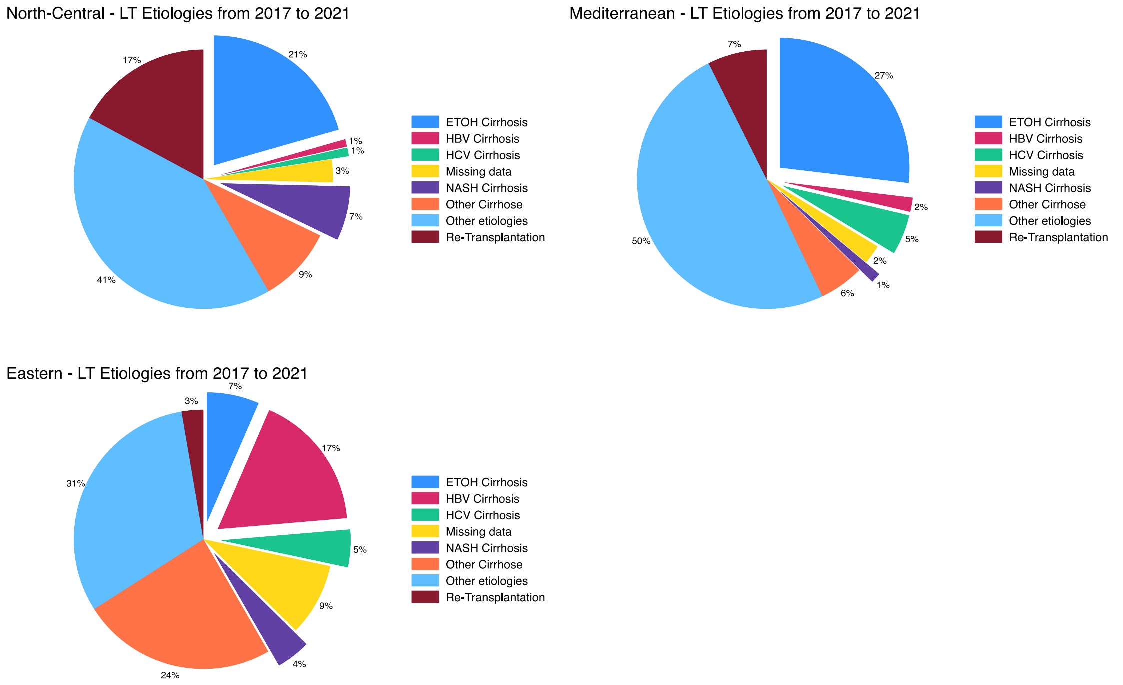

The figure shows the etiologies leading to liver transplantation across Northern, Mediterranean, and Eastern Europe. Each pie chart represents the distribution of different underlying liver diseases, including alcohol-related liver disease (ALD Cirrhosis), HBV Cirrhosis, HCV Cirrhosis, NASH Cirrhosis, and other aetiologies.

Fig. S3 – Hepatocellular Carcinoma (HCC) Trends in Liver Transplantation Across Europe

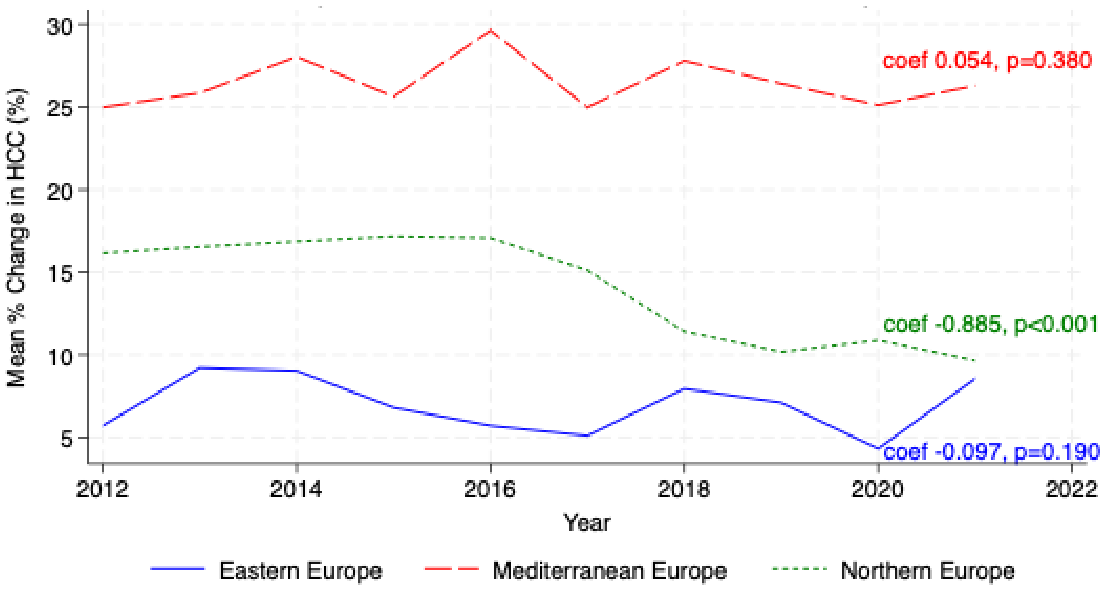

The figure shows the trends in the percentage of liver transplants performed for hepatocellular carcinoma in Eastern, Mediterranean, and Northern Europe. The lines represent the mean percentage change in HCC-related transplants over time for each region.

Fig. S4: Trends in Liver Transplantation for Non-HCC Malignancies Across Europe

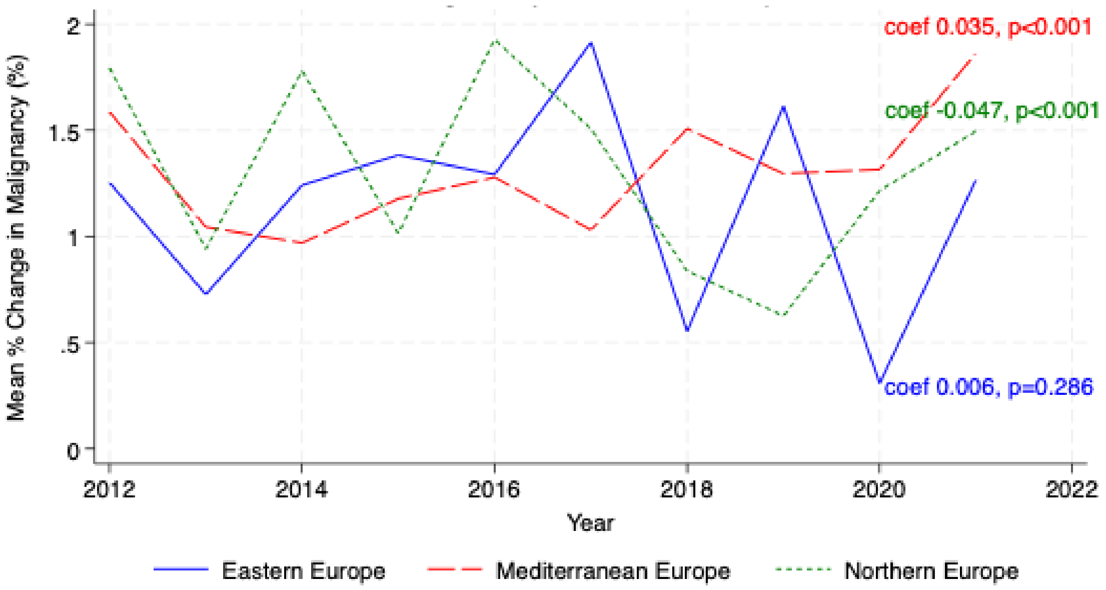

The figure shows trends in the percentage of liver transplants performed for non-HCC malignancies in Eastern, Mediterranean, and Northern Europe. The lines represent the mean percentage change in non-HCC-related transplants over time for each region.

Fig. S5. Effects of Gender on Liver Transplantation by Age, Indication, and Region

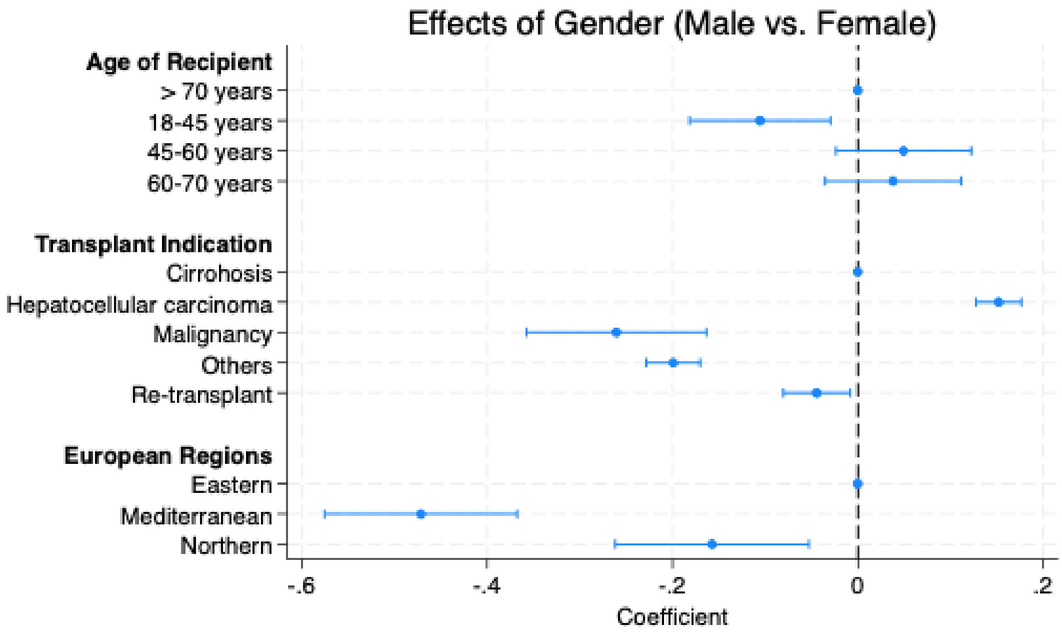

The figure shows the effects of gender (male vs. female) on liver transplantation outcomes in Europe, categorized by recipient age, transplant indication, and European region. The coefficients indicate the likelihood of being male versus female, highlighting age groups, indications, and regions where gender representation significantly differs.

Fig. S6. Trends in Liver Transplant Activity, Newly Included Patients, and Waiting List Mortality in Europe

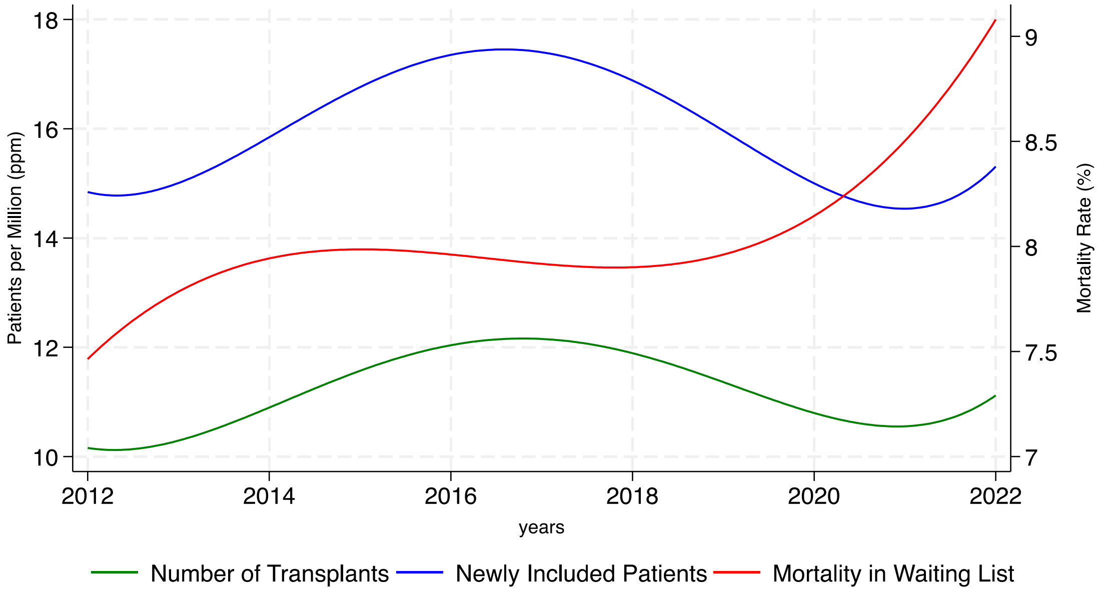

The figure shows the trends in liver transplant activity (number of transplants), newly included patients on the waiting list, and mortality on the waiting list across European countries. Trends are presented from 2012 to 2022, highlighting changes over time and the impact of factors such as the COVID-19 pandemic.

Table S1: Multivariable Analysis of Gender Representation in Liver Transplant Recipients Across European Regions

Variable	Coefficient	95% CI	p-value	Sig.
**Age Group**				
- 18-45 years	-0.106	[-0.182, -0.029]	0.007	**
- 45-60 years	0.049	[-0.024, 0.122]	0.185	
- 60-70 years	0.038	[-0.035, 0.111]	0.309	
**Transplant Indication**				
- Hepatocellular carcinoma	0.152	[0.127, 0.177]	<0.001	***
- Malignancy	-0.260	[-0.358, -0.163]	<0.001	***
- Missing	-0.014	[-0.078, 0.049]	0.656	
- Others	-0.199	[-0.228, -0.170]	<0.001	***
- Re-transplant	-0.044	[-0.080, -0.008]	0.016	**
**European Region**				
- Mediterranean	-0.472	[-0.576, -0.368]	<0.001	***
- Northern	-0.157	[-0.262, -0.053]	0.003	**

Males are more likely to undergo LT for hepatocellular carcinoma (HCC), while females are more represented in non-HCC malignancies and in Mediterranean and Northern Europe. Younger recipients (18–45 years) are more likely female, while older age groups trend male. *[Coefficients, 95% confidence intervals (CIs), and p-values are provided. Statistical significance is indicated by p-values <0.05 (*), <0.01 (**), and <0.001 (***)].*

Table S2: Healthcare and Socioeconomic Factors Influencing Liver Transplantation Practices in European Countries

Country	WL (n)	WL (pmp)	LT (n)	LT (pmp)	LT efficacy	GDP (US $)	Life Expec t. (years)	ICU beds	Hosp . beds	Alcohol (L)	(UHC)	Health Exp. (US $)	Delta Health Exp.
Austria	295	32	169	19	57	56.506,00	81	22	8	12	85	5.684,00	16%
Belgium		0	297	25		53.475,00	82	16	5	9	86	5.201,00	15%
Bulgaria	66	10	6	1	9	1.797,00	74	32	8	12	73	2.233,00	63%
Croatia	165	40	89	22	54	21.459,00	78	11	6	8	80	2.353,00	42%
Cyprus	NA	0	NA	0	NA	NA	NA	NA	NA	NA	NA	NA	NA
Czech Republic	NA	10	180	17	NA	30.427,00	79	12	7	12	84	3.559,00	46%
Denmark	85	15	46	8	54	67.967,00	81	7	3	10	82	5.052,00	10%
Estonia	21	16	9	7	43	29.823,00	78	15	4	11	79	2.550,00	57%
Finland	78	14	62	11	79	53.755,00	81	6	3	9	86	4.645,00	16%
France	3219	49	1294	20	40	44.460,00	82	12	6	10	85	5.249,00	17%
Germany	2144	26	748	9	35	52.745,00	81	39	8	12	88	6.448,00	31%
Greece	145	14	36	3	25	22.990,00	81	11	4	6	77	2.525,00	11%
Hungary	153	16	67	7	44	22.147,00	76	14	7	10	79	2.393,00	35%
Ireland	89	18	51	10	57	103.684,00	83	7	3	11	83	5.353,00	23%
Italy	2620	43	1479	25	56	38.373,00	83	13	3	7	84	3.324,00	7%
Latvia	8	4	3	2	38	23.184,00	75	10	5	13	75	2.788,00	134%
Lithuania	147	54	32	12	22	27.102,00	76	16	6	12	75	2.825,00	73%
Luxembourg	6	10	0	0	0	128.259,00	83	25	4	11	83	5.546,00	18%
Malta	NA	0	0	0	NA	NA	NA	NA	NA	NA	NA	NA	NA
Netherlands	296	17	211	12	71	62.536,00	82	8	3	9	85	5.312,00	8%

Poland	606	16	362	10	60	22.112,00	77		6	12	82	2.489,00	56%
Portugal	258	26	202	20	78	27.275,00	82	4	4	9	88	3.480,00	35%
Romania	467	25	75	4	16	18.419,00	75		7	17	78	1.941,00	116%
Slovakia	72	13	43	8	60	24.470,00	77	9	6	11	82	2.581,00	20%
Slovenia	33	16	18	9	55	32.163,00	81	6	4	10	84	3.286,00	32%
Spain	1656	35	1159	25	70	32.677,00	83	10	3	9	85	3.633,00	25%
Sweden	257	25	166	16	65	56.305,00	83	6	2	10	85	5.310,00	13%
United Kingdom	1836	27	922	13	50	48.866,00	82	7	2	11	88	4.552,00	28%

The table provides data on the number of patients included on the waiting list (WL) in 2022, both as absolute numbers (n) and per million population (pmp), as well as liver transplantation (LT) statistics. LT Efficiency (LT Eff) represents the percentage of patients included on the WL who were transplanted within the same year. NA indicates not available data.

Economic and healthcare markers, obtained for 2022 or the latest available value, include Gross Domestic Product (GDP) in US dollars, life expectancy (in years), the number of intensive care unit (ICU) beds (per 100,000 population) and hospital beds (per 1,000 population), and alcohol consumption (measured in liters per capita based on WHO classification). The Universal Health Coverage (UHC) index is a composite measure reflecting the accessibility and quality of essential health services in each country. Health expenditure is expressed in US dollars, and the Delta Health Expenditure metric represents the percentage change in health expenditure from 2012 to 2022.

Table S3: Impact of Healthcare Markers on Liver Transplant Efficiency in Europe

Healthcare Markers	Coef.	95% CI		P-value
**Univariable Analysis for LT Efficiency in 2022**				
ICU Beds per 100,000 Population	-1.638	-3.203	-0.073	0.041
Universal Health Coverage Index	3.589	0.597	6.558	0.021
**Multivariable Mixed Regression for LT Efficiency Trends (2012-2022)**				
Gross Domestic Product per capita	-0.251	-1.158	0.657	0.588
Life Expectancy	-0.449	-1455	0.558	0.382
Hospital Beds per 1,000 Population	-0.251	-0.823	0.320	0.389
Overall Health Intake	-0.075	-1427	1277	0.914
Universal Health Coverage Index	1.229	0.359	2.100	0.006
Health Expenditure in 2022	-0.204	-0.805	0.397	0.505
Relative Health Expenditure (from 2012-2022)	-3.995	-6.606	5.806	0.900
**Principal Component Analysis of Healthcare Markers Impacting LT Efficiency**				
GDP, Life Expectancy, and Healthcare Expenditure (comp1)	5.312	1.732	8.892	0.004
Hospital Beds, Alcohol Intake, and Health Expenditure (comp2)	-4.092	-8.979	0.794	0.101
ICU and Hospital Beds, Relative Health Expenditure (comp4)	-26.779	-50.038	-3.520	0.024

ICU: Intensive Care Unit; UHC: Universal Health Coverage; GDP: Gross Domestic Product.

Univariable analyses show a negative association with ICU beds (p = 0.041) and a positive association with the UHC index (p = 0.021). Multivariable regression highlights a positive effect of the UHC index (p = 0.006). Principal Component Analysis identifies a positive influence of GDP, life expectancy, and healthcare expenditure (component 1, p = 0.004) and a negative impact of ICU and hospital beds with relative health expenditure (component 4, p = 0.024).

[Coefficients, 95% confidence intervals (CIs), and p-values are provided. Statistical significance is indicated by p-values <0.05 (*), <0.01 (**), and <0.001 (***)].

Table S4: Country-Specific Policy Changes Influencing Liver Transplant Practices in Europe

Country	Year(s) of Change	Policy Change Description	Potential Impact on Transplantation Practices
Germany	2013	Stricter allocation regulations after scandals; transparency measures implemented.	Decreased liver transplants temporarily due to increased scrutiny.
United Kingdom	2018	New national liver allocation policy, introducing super-urgent categories and prioritizing urgent cases.	Improved equity and reduced waiting list mortality.
Spain	2012 onwards	Expansion of DCD programs and public awareness campaigns, optimization of organ procurement.	Increased transplantation rates and reduced waiting list mortality.
France	2013, 2017	Allocation system revised for urgent cases, with accelerated allocation for acute liver failure and high MELD patients.	Improved access for urgent patients, reduced waiting list mortality.
Italy	2015	Mandatory-split liver policy and MELD ≥ 30 prioritization adopted in 2014.	Increased use of split livers and higher urgency patients receiving faster access.
Netherlands	2016	New allocation system based on urgency and utility; expanded use of DCD donors.	Improved efficiency in organ allocation and increased transplantation rates.
Poland	2016	Centralization of transplant coordination, introduction of a national waiting list, and standardized allocation criteria.	Increased transplantation activities and efficiency.
Austria	2018	Integration of MELD score into the allocation system, adoption of extended donor criteria.	Improved fairness in allocation and increased donor pool.
Belgium	2017	Revised allocation policies prioritizing urgent cases and enhanced cooperation with Eurotransplant.	Improved access for urgent cases and pediatric patients.
Croatia	2016	Expansion of donor criteria and promotion of deceased donation.	Increased deceased donor rates and transplantation activities.

Czech Republic	2015	Centralized waiting list and allocation system introduced; expansion of DCD programs.	Improved allocation efficiency and increased utilization of DCD donors.
Denmark	2019	New allocation guidelines focusing on urgency and recipient outcomes.	Enhanced equity in organ allocation and optimization of transplant outcomes.
Sweden	2017	Revised national allocation policy to prioritize urgency, broader sharing of organs.	Enhanced equity in allocation and increased transplant activities.
Turkey	2014	Promotion of living donor liver transplantation, stricter regulations on donor safety and recipient selection.	Increased living donor transplantation rates and enhanced safety protocols.
Data for this table were obtained from public sources as detailed in the References section of the supplementary materials [1–8]			

MELD: Model for End-Stage Liver Disease; DCD: Donation after Circulatory Death. This table summarizes key policy changes in liver transplantation practices across European countries during the study period. Changes include revisions to allocation systems, expansion of donor criteria, and prioritization strategies. Notable impacts include increased transplantation rates with the introduction of DCD programs (e.g., Spain, Netherlands) and improved equity with stricter allocation regulations (e.g., Germany, United Kingdom). Data were obtained from public sources accessed in September 2024.

Supplementary references

1. *Eurotransplant*. https://www.eurotransplant.org. Accessed September 27, 2024.
2. NHS Organ Donation and Transplantation (UK). https://www.odt.nhs.uk. Accessed September 27, 2024.
3. *Organización Nacional de Trasplantes* (ONT, Spain). https://www.ont.es/. Accessed September 27, 2024.
4. *Agence de la Biomédecine* (France). https://www.agence-biomedecine.fr/?lang=fr Accessed September 27, 2024.
5. *Centro Nazionale dei Trapianti (CNT)*. https://www.iss.it/centro-nazionale-trapianti. Accessed September 27, 2024.
6. Czech Transplantation Society. https://transplant.cz/intranet/. Accessed September 27, 2024.
7. Swedish Transplantation Society. https://svensktransplantationsforening.se. Accessed September 27, 2024.
8. Ministry of Health of Turkey. https://www.saglik.gov.tr. Accessed September 27, 2024.
